# Historic hybridization and persistence of a novel mito-nuclear combination in red-backed voles (genus Myodes)

**DOI:** 10.1186/1471-2148-9-114

**Published:** 2009-05-21

**Authors:** Amy M Runck, Marjorie D Matocq, Joseph A Cook

**Affiliations:** 1 Department of Biological Sciences, Idaho State University, Pocatello, Idaho 83209, USA; 2 Department of Natural Resources and Environmental Science, University of Nevada, Reno, Nevada 89512, USA; 3 Biology Department and Museum of Southwestern Biology University of New Mexico, Albuquerque, New Mexico 87131, USA

## Abstract

**Background:**

The role of hybridization in generating diversity in animals is an active area of discovery and debate. We assess hybridization across a contact zone of northern (*Myodes rutilus*) and southern (*M. gapperi*) red-backed voles using variation in skeletal features and both mitochondrial and nuclear loci. This transect extends approximately 550 km along the North Pacific Coast of North America and encompasses 26 populations (n = 485). We establish the history, geographic extent and directionality of hybridization, determine whether hybridization is ongoing, and assess the evolutionary stability of novel genomic combinations.

**Results:**

Identification of *M. rutilus *and *M. gapperi *based on the degree of closure of the post-palatal bridge was concordant with the distribution of diagnostic nuclear MYH6 alleles; however, an 80 km zone of introgressed populations was identified. The introgressant form is characterized by having mitochondrial haplotypes closely related to the northern *M. rutilus *on a nuclear background and morphological characteristics of southern *M. gapperi*.

**Conclusion:**

Introgression appears to have been historic as pure populations of *M. rutilus *are now isolated to the north from introgressants or pure *M. gapperi *by the LeConte Glacier. As we do not find pure *M. rutilus *or *M. gapperi *individuals throughout the distribution of the introgressant form, it appears that the introgressants are a self-sustaining entity not requiring continued hybridization between pure parental forms to generate this novel combination of characters.

## Background

The evolutionary significance of hybridization has been widely recognized in some taxa such as plants, but our understanding of how this process contributes to animal diversity, especially in vertebrates is relatively limited [1-3]. Ecological and demographic settings known to contribute to hybridization between otherwise well-defined species include newly established contact (such that behavioral, pre-zygotic filters to breeding may not exist) and low density of one or both parental species such that conspecific mating opportunities are limited [4]. Such biogeographic and demographic conditions are known to characterize many areas of interspecific, post-glacial contact where the leading edges of the expanding ranges of post-glacial colonizers meet [5-8]. Such regions provide a unique opportunity to examine the origin and maintenance of hybrid forms within an increasingly well-understood biogeographic and temporal framework.

Hybrid zones between species are thought to be maintained by two primary classes of models that predict 1) differential fitness between pure parental and hybrid individuals and 2) differential degrees of spatial overlap between pure parental species. The 'tension zone' model [4] posits a dynamic balance between selection against hybrids and dispersal of hybrids into the zone while the 'bounded superiority' model [9] holds that hybrids are superior to pure parental types in a limited set of environments. As such, the tension zone model necessitates continued generation of hybrid offspring from pure parentals so such systems would be characterized by sympatry of pure parental and hybrid forms on a spatial scale that encompasses the dispersal distances of the focal species. Alternatively, if hybrids have a selective advantage, even in a limited set of environmental conditions, we would predict little to no spatial overlap between pure parental and hybrid individuals. If the distribution of hybrid-appropriate habitat is at a scale that greatly exceeds the dispersal distance of pure parentals, maintaining occupancy of these areas would require that the introgressed form become independently sustaining. It is through this latter scenario that introgressive hybridization could establish stable, evolutionarily independent populations.

A particularly common hybrid form is a pattern of mitochondrial introgression leading to novel cytonuclear combinations. Because mitochondrial DNA is maternally inherited, this pattern can exist in animals where females are the homogametic sex (i.e. mammals). Due to reproductive inferiority of the heterogametic sex (Haldane's rule), the perpetuation of such novel cytonuclear combinations is prevented in animals with heterogametic females [10]. A plausible model for a pattern of mitochondrial introgression would be one wherein interspecific hybrids initially backcross with the most available pure parental species and then become a self-perpetuating entity, perhaps even displacing pure parentals. Under such a model, introgressant hybrids would be characterized by a mitochondrial genome of one parental taxon on a genomic background composed predominantly of that of a second taxon. Phylogenetic relationships and overall levels of diversity of mitochondrial types in the new introgressant hybrid form relative to the parental taxa could provide a great deal of insight into the origin of the introgressant form as well as subsequent factors contributing to the maintenance of these novel combinations.

Where the distributions of the northern red-backed vole (*Myodes rutilus*) and southern red-backed vole (*Myodes gapperi*) meet, we have an opportunity to examine interspecific interactions at the leading edge of two expanding ranges. As the ice sheets retreated, *M*. *rutilus *expanded its distribution south from Beringia, while *M*. *gapperi *colonized northward into Canada approximately 13,500 years before present (Ka) [11,12]. Hybridization between the two species has been hypothesized based on discordance between morphological and mitochondrial traits in a limited number of field-collected specimens [13], convergence in allozyme variation in areas of contact [14] as well as successful interspecific crosses in the laboratory [15]. Despite their capacity to interbreed, *M. rutilus *and *M. gapperi *are not sister lineages and are separated by approximately 9% sequence divergence in the mitochondrial cytochrome *b *gene [16]. Our goals were to 1) establish the geographic extent and directionality of any introgression between these taxa in southeast Alaska, 2) assess whether hybridization between *M. rutilus *and *M. gapperi *is ongoing and/or historic, and 3) assess the evolutionary stability of any novel genomic combinations.

## Results

### Post-palatal bridge morphology

**Figure 1:**
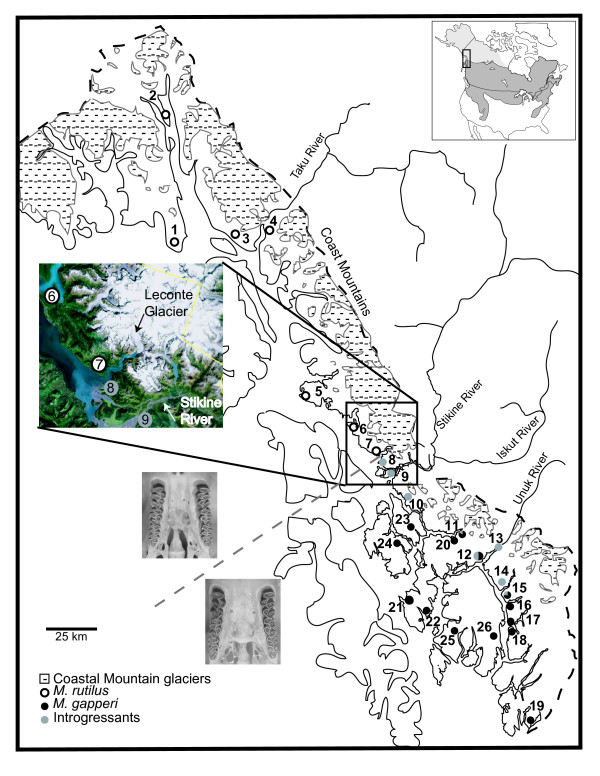
**Distribution map of *Myodes rutilus*, *M. gapperi*, and introgressants in southeast Alaska**. Open circles refer to individuals with post-palatal bridge morphology, cytochrome *b *haplotypes, and MYH6 alleles of *M. rutilus*. Black shaded circles refer to individuals with post-palatal bridge morphology, cytochrome *b *haplotypes, and MYH6 alleles of *M. gapperi*. Grey shaded circles refer to individuals with post-palatal bridge morphology and MYH6 alleles of *M. gapperi *and cytochrome *b *haplotypes of *M. rutilus*. Numbers correspond to population numbers in Table 1. The grey dashed line indicates change in post-palatal bridge morphology. Stippled areas indicate present-day glaciers. Inset of highlighted area is GIS glacial coverage that shows the LeConte Glacier extending to the coast, resulting in a physical barrier between populations 7 and 8.

There was a distinct break in character state of the post-palatal bridge near the Stikine River (Table 1). Individuals from Jap Creek (locality 7, Figure 1) and north had incomplete post-palatal bridges, which is the same character state observed in the *M*. *rutilus *from the reference sample (interior Alaska), and from throughout its range (Runck in prep). Individuals from Mallard Slough (locality 8, Figure 1) and south had complete bridges, characteristic of the *M*. *gapperi *reference sample (Minnesota) and from specimens of *M. gapperi *throughout its range (Figure 1; Runck in prep). There were, however, two individuals (localities 3 & 4), north of Mallard Slough that possessed complete post-palatal bridges, and two individuals (localities 14 & 23) south of Mallard Slough that had incomplete post-palatal bridges. In interior Alaska, 5 of 46 *M. rutilus *individuals had complete post-palatal bridges suggesting a low incidence of natural variation within this character in the northern red-backed vole.

**Table 1:** Sampling localities and number of individuals examined for morphological and molecular data

**Locality**		**N**	**Cytochrome *b***		**MYH6**		**Post-palatal bridge**	
			*rutilus*	*gapperi*	*rutilus*	*gapperi*	*incomplete*	*complete*
**Southeast Alaska *M. rutilus***								
1	Excursion Inlet	6	6	-	6	-	-	-
2	Mud Bay	20	20	-	20	-	7	-
3	Echo Cove	20	20	-	20	-	19	1
4	Limestone Inlet	8	8	-	8	-	3	1
5	Cape Fanshaw	18	18	-	18	-	4	-
6	Patterson River	13	13	-	13	-	9	-
7	Jap Creek	10	10	-	10	-	1	-
**Southeast Alaska introgressants**								
8	Mallard Slough	28	28	-	-	28	-	21
9	Stikine River	20	20	-	-	20	-	18
10	Berg Bay	5	5	-	-	5	-	3
11	Tyee	29	5	24	-	29	-	11
12	Reflection Lake	17	8	9	-	17	-	17
13	Unuk River	31	31	-	-	31	-	25
14	Chickamin River	15	10	-	-	10	1	14
15	Hut Point	16	4	12	-	16	-	15
**Southeast Alaska *M. gapperi***								
16	Ledge Point	7	-	7	-	7	-	7
17	N Rudyerd Bay	17	-	17	-	17	-	11
18	Point Louise	19	-	19	-	19	-	14
19	Gwent Cove	20	-	20	-	20	-	17
20	Duck Point	26	-	26	-	26	-	3
21	Union Bay	25	-	24	-	24	-	12
22	Bond Bay	24	-	24	-	24	-	24
23	Wrangell Island	36	-	36	-	36	1	32
24	Etolin Island	34	-	20	-	20	-	24
25	Revillagigedo Is.	12	-	12	-	12	-	9
26	Revillagigedo Is.	9	-	9	-	9	-	6
**Interior Alaska *M. rutilus***								
Denali Nat. Park		46	1	-	1	-	41	5
**Minnesota *M. gapperi***								
Brown County		18	-	1	-	1	-	18

Individuals examined for morphological and molecular data. Locality numbers correspond to Figure 1. Numbers of individuals examined for cytochrome *b *and MYH6 include direct sequencing and RFLP analyses. Contact of *M*. *gapperi *and introgressants is found at localities 11, 12, and 15.

### Nuclear gene MYH6 genetic variation

**Figure 2:**
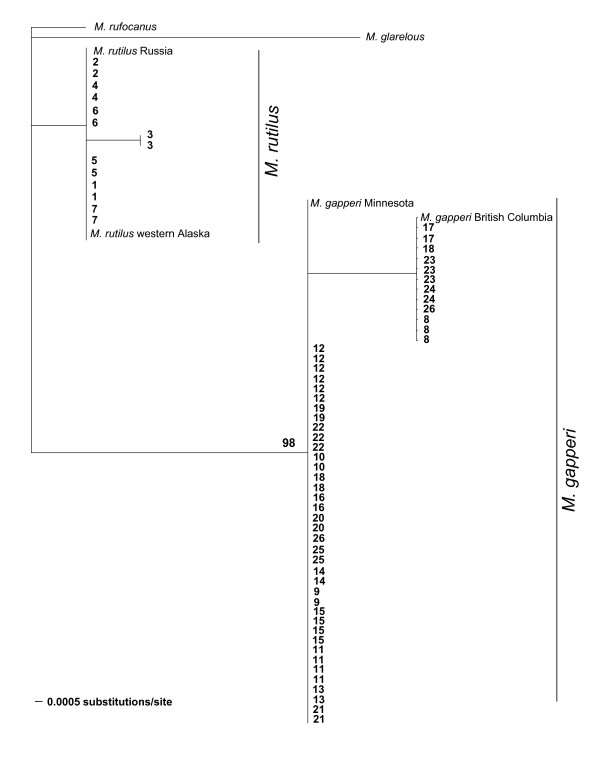
**Maximum likelihood tree constructed with 257 bp of the MYH6 locus**. Phylogeny was constructed using maximum likelihood and the HKY model of nucleotide substitution. Numbers above the branches are bootstrap support values > 65. Taxa labels are the locality number. The vertical bars show the morphological species identifications.

Phylogenetic analyses of sequences of MYH6 revealed two clades with an average uncorrected pairwise divergence of 1.94% (Figure 2). Maximum likelihood, neighbor-joining, and Bayesian analyses produced similar topologies. Individuals from Jap Creek (locality 7) and northward had alleles that formed a clade with *M. rutilus *from western Alaska and Russia (Figure 2). Individuals from Mallard Slough (locality 8) and southward had alleles that formed a clade with *M. gapperi *from Minnesota and British Columbia. Uncorrected sequence divergence between the outgroup taxa and the *M*. *gapperi *and *M*. *rutilus *clades were 2.98% and 1.80%, respectively. None of the sequenced individuals was heterozygous at the diagnostic nucleotide positions.

Further sampling of the species-specific SNP using RFLP analyses resulted in the digestion pattern diagnostic of *M. rutilus *in all individuals from Jap Creek (7) northward, while all individuals from Mallard Slough (8) southward had MYH6 alleles diagnostic of *M. gapperi*. We found no individuals heterozygous for species-specific alleles.

### Mitochondrial genetic variation and character concordance

We found 46 unique mitochondrial cytochrome *b *haplotypes in our entire sample. Patterns of variation across haplotypes were characteristic of functional mitochondrial genes, with average base frequencies of A (29.3) G (13.5) C (29.6) T (27.6), a 3.64 transition/transversion ratio, and a gamma distribution of 0.75 of changes across classes of codon sites. In addition, the distribution of the variable amino acid residues was consistent with the model of variable and conserved regions in cytochrome *b *[17].

**Figure 3:**
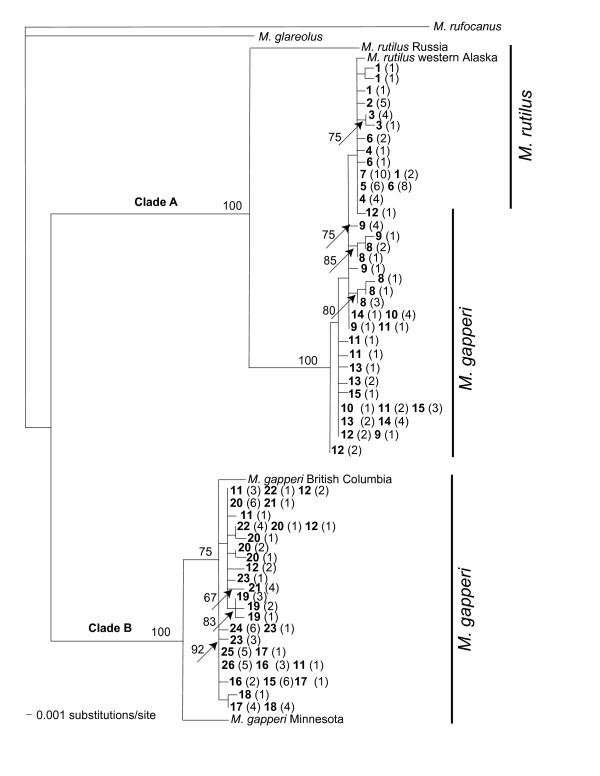
**Maximum likelihood tree constructed with 600 bp of the cytochrome *b *gene**. Phylogeny was constructed using the TrN + invariable sites + gamma model of nucleotide evolution. Number above the branches are bootstrap support values > 65. The taxa labels in bold are the locality number and the numbers in parentheses are the number of individuals. The vertical bars indicate the morphological species identifications.

Phylogenetic reconstruction using maximum likelihood, Bayesian, and neighbor-joining methods produced similar topologies consisting of two highly supported clades, A and B (bootstrap support of 100; Figure 3). Average pairwise sequence divergence between southeast Alaska individuals in clades A and B is 7.2% (uncorrected *p *distance). Clade A haplotypes extended from Siberia, through western Alaska, and along the southeast Alaskan coast north of Hut Point (15; Figure 3). Clade B haplotypes were found in Minnesota and British Columbia as well as the southeast Alaskan coast from Hut Point (15) southward to Gwent Cove (19) on the mainland, Tyee (11) to Bond Bay (22), on the Cleveland Peninsula, and Wrangell (23), Etolin (24), and Revillagigedo (25, 26) islands. Through additional RFLP screening of cytochrome *b*, we determined that all individuals north of the Stikine River (localities 1–8) and individuals up to 80 km south of the Stikine River (localities 9 – 15) had Clade A mitochondrial haplotypes. Clade B haplotypes were found on the mainland south of Hut Point (localities 15 – 19), on the Cleveland Peninsula (localities 11, 12, 20 – 22) and on Wrangell (locality 23), Etolin (locality 24), and Revillagigedo islands (localities 25, 26). Contact between the two divergent haplotype clades were found at localities 11, 12, and 15.

Individuals with Clade A haplotypes that were also characterized by the post-palatal bridge morphology of *M. rutilus *and MYH6 alleles concordant with this morphology were only found north of Jap Creek (7). Hereafter, we refer to these individuals as *M. rutilus*. Individuals with Clade B haplotypes and the post-palatal bridge morphology of *M. gapperi *and MYH6 alleles concordant with this morphology were found on Etolin (23), Wrangell (24), Revillagigedo (25, 26) islands, the Cleveland Peninsula (20 – 22), and south of Hut Point (15), (hereafter *M. gapperi*). All individuals from Mallard Slough (8) south to Berg Bay (10) and Unuk and Chickamin rivers (13 & 14) had the post palatal bridge morphology and MYH6 sequence characteristic of *M. gapperi *but a set of cytochrome *b *haplotypes more closely related to pure *M. rutilus *(hereafter introgressants; Figure 1). Introgressants were found in sympatry with *M*. *gapperi *only at localities 11, 12, and 15 (Figure 1; Table 1). Though haplotypes found within the introgressant individuals as a group were most closely related to *M. rutilus*, none was identical to haplotypes found in populations of *M. rutilus*. A synapomorphic mutation (position 513) was shared by all but one individual (Reflection Lake; locality 12) of the 45 sequenced introgressants.

**Figure 4:**
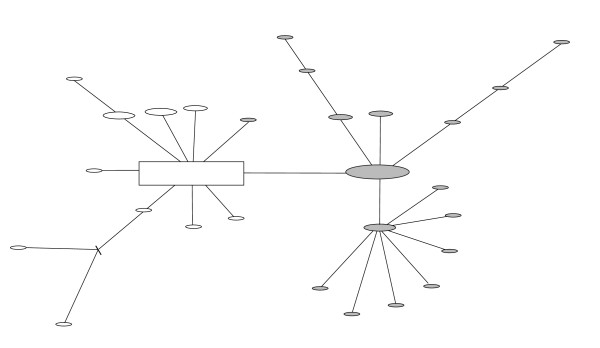
**Cytochrome *b *gene genealogy of Clade A haplotypes**. Statistical parsimony network was constructed using all unique Clade A haplotypes with the inferred ancestral haplotype indicated by the square. The size of the squares and ovals correspond to the haplotype frequencies. Haplotypes represented by white ovals (n = 11) were found in individuals identified as *M. rutilus *and haplotypes represented by grey ovals (n = 18) were found in individuals identified as introgressants. A hash mark indicates intermediate mutational steps. The parsimony criterion was met, as the number of mutational steps between any pair of haplotypes was ≤ 7.

**Figure 5:**
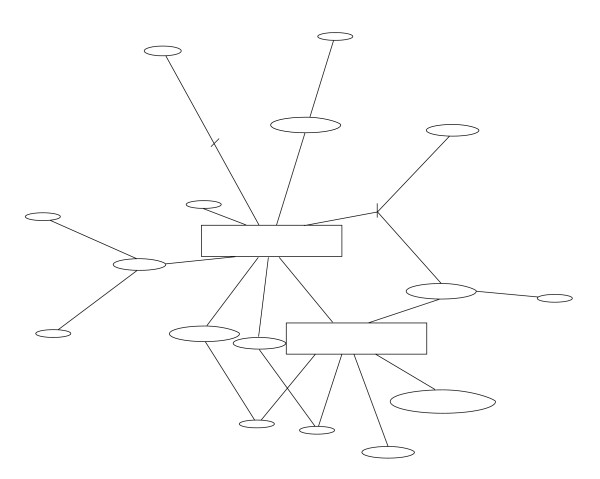
**Cytochrome *b *gene genealogy of Clade B haplotypes**. Statistical parsimony network was constructed using all unique Clade B haplotypes. These haplotypes were found in individuals identified as *M*. *gapperi*. Inferred ancestral haplotypes are represented by squares. The size of the square and ovals correspond to the haplotype frequencies. A hash mark indicates intermediate mutational steps. The parsimony criterion was met, as the number of mutational steps between any pair of haplotypes was ≤ 5.

To further resolve haplotype relationships within the two major clades, we constructed statistical parsimony networks using cytochrome *b *sequence variation without violating the parsimony criterion, as haplotypes within each clade were ≤ 10 mutational steps away from each other. The Clade A network had two major subclades, one consisting of haplotypes only found in the introgressant form (Figure 4; indicated in grey) and the other consisting of all pure *M*. *rutilus *individuals and one introgressant (Figure 4). The maximum distance between pure *M*. *rutilus *individuals was five mutations (0.008%) and between introgressant individuals six mutations (0.01%). Two ancestral haplotypes were inferred in the Clade B network (internal nodes) and a maximum distance of 5 mutations (0.008%) was found among these *M. gapperi *haplotypes (Figure 5).

Haplotype diversity was highest in *M*. *gapperi *(0.906 +/- 0.01) and in the introgressants (0.874 +/- 0.04) and lowest in *M*. *rutilus *(0.633 +/- 0.07; Table 2). Likewise, nucleotide diversity was highest in *M*. *gapperi *and the introgressants (0.003 +/- 0.002) and lowest in *M*. *rutilus *(0.001 +/- 0.001). Even though the introgressant haplotypes are nested within *M*. *rutilus*, haplotype and nucleotide diversities in *M*. *rutilus *and the introgressants are significantly different t_√90 _= 18.22 p = < 0.001 and t_√90 _= 31.96 p = < 0.001, respectively.

**Table 2:** Cytochrome *b *summary statistics for *M. rutilus*, *M. gapperi *and introgressants

**Group**	**N**	**H**	**S**	***h ***± SD	**π **± SD	**Fu's Fs**	τ
***M. rutilus***	47	11	11	0.633 ± 0.07	0.001 ± 0.001	-7.8*	0.97 (0.48 – 1.51)
***M. gapperi***	80	18	16	0.906 ± 0.01	0.003 ± 0.002	-8.9*	2.13 (1.78 – 2.70
**Introgressants**	45	17	17	0.874 ± 0.04	0.003 ± 0.002	-11.3*	1.93 (1.32 – 2.69)

Diversity measures from the analysis of cytochrome *b *sequences. Number of individuals examined (N), number of haplotypes (H), number of segregating sites (S), gene diversity (*h*), nucleotide diversity (π), Fu's Fs, for *M. rutilus, M. gapperi*, and introgressants. Estimates of time since expansion in mutational units (τ) are calculated from the mismatch distribution with their 95% confidence intervals.

* Significant p < 0.01.

### Demographic history and molecular evolution

**Figure 6:**
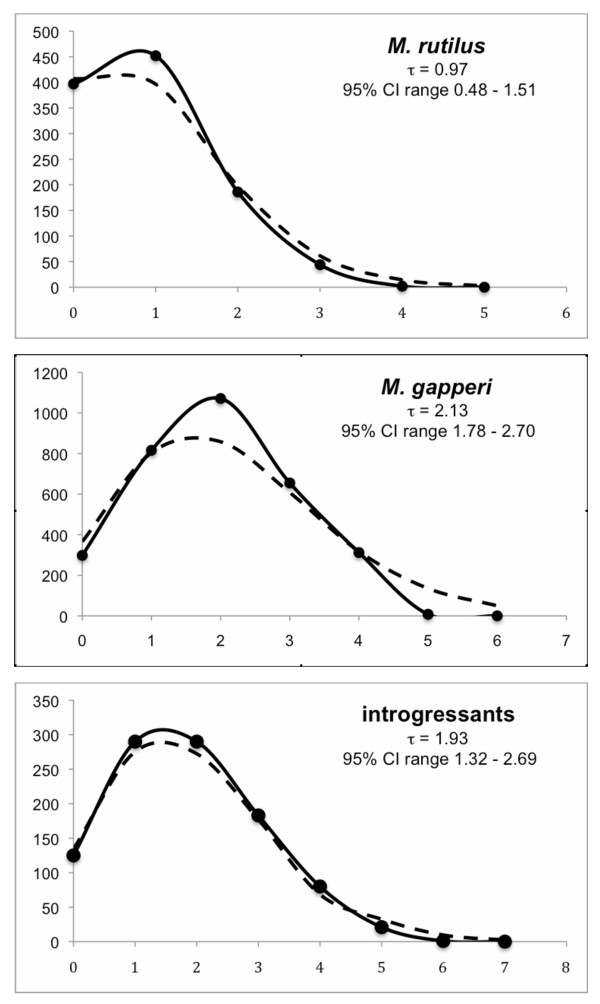
**Mismatch distribution**. Mismatch distribution for *M. rutilus*, *M. gapperi *and introgressants. The solid and dashed lines indicate observed and expected distributions, respectively.

Values obtained through Fu's test of selective neutrality were largely negative and significantly different from zero (Table 2), which is expected for populations undergoing recent growth. However, negative values can also be a result of selection. The mismatch distributions were unimodal for *M*. *rutilus*, *M*. *gapperi*, and the introgressants, which is expected for populations undergoing sudden expansion or under certain selective regimes (Figure 6).

Estimates of the timing of any expansion events (τ) were very similar in *M*. *gapperi *and the introgressants, but more recent in *M*. *rutilus *(Table 2). The unimodal peak of the mismatch distributions (τ) were used to calculate time since expansion (t = τ/2u), which we estimate to be 9.46 Ka (95% CI range 7.91 – 12.0 Ka) for *M*. *gapperi *and 8.57 Ka (95% CI range 5.86 – 11.95 Ka) for the introgressants. Estimate of time since expansion is most recent in *M*. *rutilus *at 4.31 Ka (95% CI range 2.12 – 6.74 Ka).

Relative rate tests were conducted using pairwise lineage comparisons and *M*. *rufocanus *as an outgroup. Because of saturation, rates of synonymous substitutions per synonymous site (Ks) could not be calculated, so rates of synonymous transversions per fourfold degenerate site were estimated (B4), as the rate of evolution in transversions is slower. Pairwise lineage comparisons of B4 showed no significant difference among the rates of evolution (p > 0.27). Rates of non-synonymous substitutions per nonsynonymous site (Ka) were also similar among the lineages (p > 0.30; results not shown). The molecular clock hypothesis was not violated, as likelihood scores of trees constructed with and without molecular clock constraints were not significantly different (χ^2 ^= 44.8 d.f. = 176, P > 0.05).

## Discussion

After the retreat of Pleistocene glaciers, distributions of many high latitude organisms shifted resulting in new species assemblages and opportunities for genetic and ecological interactions [5-8,18]. Historical genetic signatures of heterospecific mitochondrial genes may be preserved in hybrid zone populations that are no longer undergoing genetic exchange. Examination of these populations using population and coalescent methods should provide insight into the timing and dynamics of geographical shifts in species' ranges in response to climate change [8,19].

### Concordance of diagnostic characters

Identification of *M. rutilus *and *M. gapperi *in this transect based on the degree of closure of the post-palatal bridge was concordant 98.7% of the time with the distribution of the species-specific MYH6 alleles, with the exception of four individuals. The discordance observed in these individuals was a result of the character state of the post-palatal bridge being opposite of what was found in all other members of their respective population. We therefore reason that because there is a distinct change in the developmental state of the post-palatal bridge at the Stikine River area and this character distinguishes *M. rutilus *from *M. gapperi*. This abrupt change in frequency of the ossification of post-palatal bridge and distribution of the MYH6 diagnostic alleles coincides with Hall's [20] depiction of parapatry of *M. rutilus *and *M. gapperi *at the Stikine River area.

The distribution of highly differentiated mitochondrial cytochrome *b *haplotypes was not concordant with the morphological and nuclear characters. Our analysis revealed three groups: *M. rutilus *is characterized by an incomplete post-palatal bridge and a set of closely related MYH6 alleles and cytochrome *b *haplotypes; *M. gapperi *is characterized by a complete post-palatal bridge and a set of closely related MYH6 alleles and cytochrome *b *haplotypes that are highly differentiated from those of *M. rutilus *(1.9% and 7.2%, respectively); and an introgressant form that has the post palatal bridge and MYH6 alleles of *M. gapperi *but a set of cytochrome *b *haplotypes that is unique, yet clearly more closely related to *M. rutilus *haplotypes. Thus, across an 80 km expanse separating pure *M. rutilus *and *M. gapperi *populations, these introgressant red-backed voles are characterized by a combination of features of both *M. rutilus *and *M. gapperi*.

### Colonization and hybridization dynamics

Estimates of expansion times into southeast Alaska obtained from the mismatch distribution (t = τ/2u) indicate that these species arrived post-glacially. Working under the assumption that rates of evolution are consistent among these three groups, estimates of time since expansion are similar in *M*. *gapperi *and the introgressants, dating to 9.46 Ka and 8.57 Ka, respectively. As introgressants largely reflect the genetic signature of the hybridizing *M*. *rutilus*, expansion of both species into southeast Alaska date back to the early Holocene [21,22]. These estimates of expansion into southeast Alaska are notably earlier than the expansion time of those pure populations of *M. rutilus *that now exist north of the LeConte Glacier (4.31 Ka).

Given our refined view of the distribution of *M. rutilus *and *M. gapperi *and potential barriers, there appears to be no contemporary contact between these species in this transect. Consistent with that view, is the lack of MYH6 heterozygotes with alleles diagnostic of pure *M. rutilus *and *M. gapperi *that might suggest ongoing hybridization with *M*. *rutilus *[23]. Likewise, inspection of the post-palatal bridge, presumably controlled by multiple nuclear loci, revealed no intermediate morphs.

In addition to not finding evidence for contemporary gene exchange between these two species, we did not find the parental species in sympatry. Furthermore, there was not extensive overlap of the introgressants with the pure parentals. *M. gapperi *and the introgressants occur in sympatry only at the southern edges of the zone of introgressants (localities 11, 12, 15), and the current tidewater position of the LeConte Glacier prevents contemporary contact of pure *M. rutilus *with voles in areas farther south that are now occupied by introgressants or, even farther south, by pure *M. gapperi*. As such, it would appear that the introgressants are self-sustaining populations and not hybrids that are continuously generated from pure parental crosses. An active contact zone does exist, however, at the southern, leading edge of the introgressant distribution and the northern edge of pure *M. gapperi *(at localities 11, 12, 15). The direction and degree of genetic exchange at the latter contact zone is the subject of an ongoing study (Runck et al., in prep). Future ecological studies should explore whether abiotic or biotic shifts are associated with the transition between these groups and/or whether direct competitive interactions limit their coexistence.

### Origin of mitochondrial signature in introgressants

The introgressant form is characterized by a monophyletic group of mitochondrial haplotypes nested within haplotypes otherwise characteristic of *M. rutilus*. Nonetheless, the introgressed group possesses a distinct mitochondrial genetic signature from *M*. *rutilus *populations across this region. Notably, not only do the introgressants not share any haplotypes with *M*. *rutilus*, they also have significantly greater mitochondrial variability than the donor species.

Three plausible scenarios could lead to this distinct genetic signature of novel, but closely related, haplotypes and overall higher mtDNA diversity that characterize the introgressants. First, increased mitochondrial diversity in the introgressants may simply reflect differences in population history when compared to *M*. *rutilus *in southeast Alaska. The higher estimates of mitochondrial diversity in the introgressants are similar to those for other populations of *M*. *rutilus *outside of southeast Alaska. For 54 *M*. *rutilus *found across a much greater geographic sampling area in northwestern Canada and interior Alaska, estimates are comparable to the level of diversity seen in the introgressants, with nucleotide diversity of 0.003, 19 segregating sites, and τ = 1.63 (unpublished data). Therefore, it is possible that contemporary populations of *M*. *rutilus *in southeast Alaska previously had higher levels of diversity, but lost diversity (e.g., through bottleneck events) after hybridizing with *M*. *gapperi*. This hypothesis does not explain the lack of shared haplotypes between the introgressants and *M. rutilus*, however, so southeast Alaskan *M*. *rutilus *subsequently must have lost all the haplotypes now found exclusively in the introgressants.

A possible alternative is that southeast Alaska may have served as a glacial refugium during the Last Glacial Maximum for red-backed voles. Under this scenario, a more diverse population of *M*. *rutilus *originally hybridized with *M*. *gapperi *thus creating the introgressants, which captured and sustained *M. rutilus *haplotype diversity. As glaciers retreated, genetic diversity was lost from coastal populations of pure *M*. *rutilus *as they expanded northward (reflected in low mitochondrial variation in contemporary populations of *M*. *rutilus*). Although southeast Alaska has been proposed as a coastal refugium for vertebrates during Pleistocene glacial advances [24,25], we do not believe this is the case for northern red-backed voles again, because no introgressant haplotypes are shared with contemporary populations of *M*. *rutilus*.

A hypothesis that seems most consistent with the available data implicates multiple waves of colonization of *M*. *rutilus *into this coastal region. Multiple colonization events of *M*. *rutilus *into the region could also lead to this pattern of distinct haplotypes in the introgressants. Under this scenario, gene exchange between the species occurred first with an early colonizing population of *M*. *rutilus*, and the genetic signature of the introgressants now reflects this ancestral exchange along with the subsequent accumulation of new haplotypes through time. Later, a second colonization event would have given rise to extant *M. rutilus *in the region. If these extant-pure *M*. *rutilus *are indeed the result from a second, more recent colonization event, as the estimate of expansion suggests (~4.3 Ka), these voles would have been isolated from introgressant populations south of Jap Creek (locality 7) due to the advancement of the LeConte Glacier to tidewater around 5,000 ybp. Therefore, genetic exchange between the introgressants and contemporary *M*. *rutilus *would have been prevented and would account for the lack of shared cytochrome *b *haplotypes and lack of MYH6 heterozygotes. One would have to posit that the earlier colonizing wave of *M*. *rutilus *was extirpated from the region or was not sampled in this study.

Regional extinctions and multiple colonizations have been documented in southeast Alaska during times of climate oscillations [26] as the North Pacific Coast underwent repeated climatic fluctuations during the Pleistocene and Holocene. Recent advances correspond to the Younger Dryas around 10.6-9.9 Ka [27], with three additional advances around 5–6 Ka, 3.5-2.5 Ka, and 200-100 YBP [28-30]. During these cooling events, alpine glaciers advanced into lower elevations, reducing species ranges. Due to the extensive glacial coverage of the northern part of southeast Alaska, *M. rutilus *may have been susceptible to extirpation or displacement during periods of cooling and advancing glaciers.

Marginal support for the multiple waves of colonization of *M*. *rutilus *hypothesis is reflected in the relationship of the introgressant mtDNA relative to pure *M*. *rutilus *in the minimum spanning network and likelihood tree. In both analyses, introgressants form a subgroup, and are not intermixed with the remaining *M*. *rutilus *mitotypes suggesting that these two mitotypes were not part of a panmictic population. The genetic footprint of an earlier hybridization event supports the hypothesis of multiple colonizations of the northern red-backed vole along the coast.

### Evolution of an introgressant contact zone

Although genetic exchange may have been extensive, our data suggest that a very closely related set of haplotypes (or a single haplotype that subsequently mutated) was captured and maintained in the introgressants. The unimodal distribution of pairwise comparisons and position of the introgressant haplotypes in the network and phylogeny support the hypothesis of a single hybridization event instead of multiple temporally discrete events. Initial genetic exchange may also have been bidirectional, but we only have evidence thus far of the mitochondrion of *M. rutilus *being maintained on the morphological and presumably nuclear background of *M. gapperi*. Nonetheless, a more complete view of the nuclear composition of the introgressant form relative to the pure parental forms will provide insight to the extent of backcrossing that occurred in this system.

Once established, the novel mito-nuclear combination of the introgressants either diffused neutrally or was selected for and expanded their distribution while displacing the pure parentals. While we cannot determine which scenario is responsible for the 80 km zone of introgressants, it is notable that all individuals in this region are introgressants. Moreover, several of our findings are consistent with predictions of the bounded superiority hypothesis [9]. The introgressants and pure parentals do not overlap extensively and the introgressants are self-sustaining and are not the result of continual hybridization. Also, consistent with the bounded superiority hypothesis is that the introgressants occupy a limited area of southeast Alaska, and have not established populations west of localities 11 and 12 on the Cleveland Peninsula.

The degree of genetic admixture that may have occurred while the introgressant voles were fairly uncommon relative to pure parentals awaits our more complete sampling of the nuclear genome of this group. Similar long-term persistence and spatial expansion of introgressant or hybrid forms has been documented in snails (genus *Cerion*), resulting from an ancient hybridization event between a now extinct fossil species and an extant species [31]. Hybrids are hypothesized to have persisted due to the novel genetic combinations that enhanced survival during the time that one of the parental species was eliminated [31].

With the advance of the LeConte Glacier to tidewater approximately 5,000 years ago, any potential for gene flow between northern *M*. *rutilus *and the introgressants ceased. However at the southern edge, we do find localities (11, 12, and 15) where introgressants overlap spatially with pure *M. gapperi *suggesting the potential for ongoing gene flow. Ongoing analyses of microsatellite genetic variation should identify not only the degree of overall distinction between the introgressed form and pure *M. gapperi *but also the amount of gene flow that characterizes their current contact zone. Likewise, ecological studies may help identify the degree to which these groups compete either directly or indirectly with one another and whether the spatial distribution of each is currently stable or actively shifting.

### A North Pacific Coast suture zone

The North Pacific Coast has been documented as post-glacial contact zone for several mammalian lineages from the high latitude refugium called Beringia and from multiple refugia that existed south of the continental ice sheets [13]. Within species, independent colonizations into this recently deglaciated region have occurred by at least two divergent lineages of several species such as dusky shrew (*Sorex monticolus*) [32], long-tailed vole (*Microtus longicaudus*) [33], black bear (*Ursus americanus*) [34], and marten (*Martes americana*) [35,36]. Ermine (*Mustela erminea*) are represented by three divergent lineages in southeast Alaska; one is hypothesized to be endemic to the region, perhaps surviving in the North Pacific Coast during the Pleistocene [24].

Introgression along the coast has been documented from divergent lineages of marten [35], black bears [37], and now red-backed voles. Contact zones are likely for divergent clades of dusky shrews and long-tailed voles, therefore the narrow strip of mainland along the southeast Alaska coast may be a suture zone, whereby several formerly isolated species have entered the region by discrete colonization routes, and have subsequently come into contact in the same geographic area [38-40]. The North Pacific Coast, and in particular, southeast Alaska, possess characteristics [38] commonly tied to suture zones, such as being located between Pleistocene glacial refugia, and nearby low mountain passes acting as corridors for dispersal [13,41] during warm periods.

A renewed interest has emerged in testing the validity of Remington's thirteen North American suture zones through phylogeographic studies [39,40,42]. The North Pacific Coast was not originally identified as a suture zone by Remington [38], but phylogeographic studies repeatedly demonstrate the existence of multiple lineages within species (e.g., shrews, voles) in this region. Populations representing divergent lineages are now in contact there following postglacial expansion [43]. Most of these studies, however, have limited ability to detect hybridization because only mitochondrial genes were assessed. Future phylogeographic studies of these species should employ multiple independent characters to more rigorously assess the influence of geologic and climatic events on structuring diversity along the North Pacific Coast.

## Conclusion

Interspecific hybridization between *M. rutilus *and *M. gapperi *resulted in the formation of an introgressant group that spans 80 km. The novel mito-nuclear combination of these introgressants likely expanded either by displacing or by colonizing areas left unoccupied by pure parentals in response to the changing climate of the Holocene. Hybridization between the two species is historical, as a region occupied exclusively by the introgressants now separates these two species. Additionally, physical separation (as a result of glacial advances) of *M. rutilus *and introgressant populations occurred ca. 5,000 Ka thus establishing reproductive isolation of this pure parental species and the introgressants. These introgressive populations appear to be stable as they are not a result of continuous interspecific matings between the pure parental species.

## Methods

### Sampling

The temperate rain forest of coastal southeast Alaska is naturally fragmented by extensive icefields, fjords, and six major rivers, and is isolated from continental North America by the St. Elias and Coast Mountains. Sampling was conducted at 26 localities [44] spanning approximately 550 km along the North Pacific Coast centered on the Stikine River where *M. rutilus *and *M. gapperi *are traditionally depicted as being in contact [20] (Figure 1). Specimens were deposited at the University of Alaska Museum of the North. A total of 485 individuals (see Additional file 1) were analyzed, with 5 to 36 individuals from each locality. Additional individuals of *M. rutilus *and *M. gapperi *from localities outside southeast Alaska were included for comparative purposes.

### Morphological data collection

**Figure 7:**
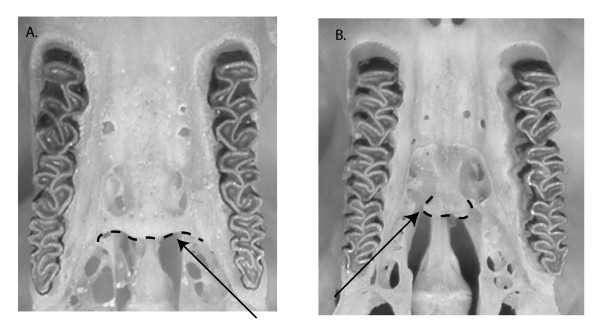
**Variation in ossification of the post-palatalbridge**. Pictures of ventral views of craniums with arrows pointing to the medial shelf. A. Complete post-palatal bridge, with the dashed line highlighting the medial shelf connected to lateral parts of the palate; diagnostic of *M. gapperi*. B. Incomplete post-palatal bridge, with the dashed line highlighting the medial shelf, which is not connected to lateral parts of the palate; diagnostic of *M. rutilus*.

Closure of the post-palatal bridge is the key diagnostic character used to distinguish *M. rutilus *from *M. gapperi *(Figure 7) [11,20,45,46]. All individuals from the transect were examined, but in some instances the character state could not be determined due to skull damage, resulting in a total 328 individuals analyzed. Character states were compared with *M*. *rutilus *skulls from interior Alaska (n = 46) and *M*. *gapperi *skulls from Minnesota (n = 18). Previous analyses of these specimens and others show that degree of post-palatal closure is not correlated with sex, age, or latitude, (Runck in prep). Specimens were examined under 25× magnification to score the post palatal bridge either as A) complete, with medial shelf connected to lateral parts of the palate, or B) incomplete, with medial shelf disconnected (Figure 7).

### Molecular methods

Genomic DNA was extracted from frozen liver of 95 *M. rutilus *and 390 *M. gapperi *following a modified salt extraction method [24,47].

### Nuclear locus, MYH6

Twenty-seven nuclear genes were screened from the collection of comparative anchor tagged sequences to obtain a diagnostic nuclear perspective on species identification and to test for interspecific gene flow in the contact zone [48]. Five loci yielded PCR products in the 300–1000 bp range. We used the primer set MYH2F and MYH2R to generate a 257 bp fragment of MYH6 (myosin heavy polypeptide 6). Although this primer set was originally designed to amplify MYH2 (myosin heavy polypeptide 2) [48], a nucleotide BLAST search against the entire nucleotide collection in GenBank indicated that the amplified fragments are MYH6.

The identification of species-specific alleles was conducted using broadly distributed individuals of *M*. *rutilus *from Russia, Finland, and western Alaska and individuals of *M*. *gapperi *from Washington, Minnesota, and North Carolina. Outgroup taxa *M. rufocanus *and *M. glareolus *were also sequenced [16]. From these samples we found three conserved single nucleotide polymorphisms (SNPs) that distinguish the alleles of *M*. *rutilus *and *M*. *gapperi*. We consider this locus to be diagnostic for the two species because these diagnostic nucleotide differences were found throughout the species' ranges.

The MYH6 locus was amplified for all individuals (n = 485). Restriction length polymorphism (RFLP) analysis of the MYH6 locus was used to delimit the distribution of species-specific alleles among individuals. A restriction site is present at one of the SNPs, and the restriction enzyme Hpa II was used to digest MYH6 amplicons. Hpa II cuts the restriction site CCGG, which is present in *M. gapperi *at positions 182 – 185, but not in *M. rutilus*. Restriction enzyme digestion was completed using 5 μl of the PCR product, 7.5 units Hpa II, and 5 μl Buffer1 (New England Biolabs). PCR products were incubated at 37°C for 5 hours with negative and positive controls. Digested products were run on 2% agarose stained with ethidium bromide.

Directing sequencing of MYH6 was conducted on at least 2 individuals from each sampling locality (n = 64; Additional file 1; GenBank FJ638345–FJ638410). Qiagen Qiaquick purification kits were used to purify PCR products. Purified PCR products were cycle sequenced using Taq DyeDeoxy Terminator Cycle Sequencing Kit and Big Dye Terminator Cycle Sequencing Ready Reaction Mix 3.0 (Applied Biosystems Incorporated). Sequencing products were filtered using Sephadex G-50 in Centrisep spin columns (Princeton Separations). Automated sequencing of both heavy and light strands was conducted on Applied Biosystems Incorporated 373 and 3100 DNA sequencers. Sequences were aligned and compared manually using Sequence Navigator, Version 1.01 (Applied Biosystems Incorporated) and Sequencher (GeneCodes). Heterozygous sites were given standard DNA degenerate codes [23].

### Mitochondrial locus, cytochrome *b*

PCR was used to amplify the first 632 base pairs of the cytochrome *b *gene with the primers MVZ 05 [49] and CLETH 06 [50] following established protocols [50]. Cytochrome *b *fragments were used in subsequent restriction enzyme digestions and direct sequencing.

Direct sequencing of the cytochrome *b *gene fragment was conducted on at least five individuals from each sampling locality (Additional file 1), resulting in a total of 47 sequences of *M. rutilus *and 125 of *M. gapperi *(GenBank FJ616001–FJ616166). Additional sequences were generated from one *M. rutilus *from Russia and one from western Alaska, and from one *M. gapperi *from British Columbia. Cytochrome *b *PCR products were purified using polyethylene glycol (PEG) precipitation [51]. Purified PCR products were cycle sequenced using Taq DyeDeoxy Terminator Cycle Sequencing Kit and Big Dye Terminator Cycle Sequencing Ready Reaction Mix 3.0 (Applied Biosystems Incorporated). Sequencing products were filtered using Sephadex G-50 in Centrisep spin columns (Princeton Separations). Automated sequencing of both heavy and light strands was conducted on Applied Biosystems Incorporated 373 and 3100 DNA sequencers. Sequences were aligned and compared manually using Sequence Navigator, Version 1.01 (Applied Biosystems Incorporated) and Sequencher (GeneCodes).

RFLP analysis was conducted on all individuals (n = 485) to determine the distribution of *M. rutilus *and *M. gapperi *cytochrome *b *gene haplotypes within the 26 populations. The ALU I restriction site AGCT is present in *M. rutilus *at position 513 but not in *M. gapperi *[16,50]. Cytochrome *b *fragments were digested using 3.5 units of ALU I, 1.0 ul Buffer2 (New England BioLabs), and 5.0 ul PCR product. Positive and negative controls were included to confirm enzyme activity in each trial. PCR products were digested for 5 hours at 37°C and then visualized on a 2.0% agarose gel stained with ethidium bromide. Digestion of the cytochrome *b *fragment resulted in two bands for *M. rutilus *and one band for *M. gapperi*. Distributions of the two haplotypes were mapped.

### Phylogenetic analysis

We analyzed 257 bp of the MYH6 locus using maximum likelihood and neighbor-joining algorithms in PAUP*b10 [52] and Bayesian statistics in Mr. Bayes v3.4 [53]. Individuals of *M. rutilus *from western Alaska and Russia and individuals of *M. gapperi *from Minnesota and British Columbia were also included in the analyses. *M*. *rufocanus *and *M*. *glareolus *sequences were included as outgroups. The model HKY [54] was determined as the simplest model that best fit these data through Modeltest [55] and was used in the analyses. Neighbor-joining analyses used 1000 bootstrap replicates to assess nodal support. The Bayesian analysis started with a random tree and was run for 1.0 × 10^7 ^generations sampling every 1,000 generations. A consensus of three runs was computed after stationarity was reached.

The first 600 base pairs of cytochrome *b *from 172 southeast coastal individuals were used in phylogenetic analyses. *M. rutilus *from Russia and western Alaska and *M. gapperi *from British Columbia were included in addition to a published sequence from Minnesota [GenBank:AY952173] [50]. *M*. *rufocanus *[GenBank:AY309416] [16] and *M*. *glareolus *[GenBank:AF119272] [56] were included as outgroups. The Tamura-Nei [57] model of nucleotide evolution with invariable sites (0.4036) and gamma distribution (0.7468) was identified using Modeltest as the model that best fit these data, and was used in subsequent analyses. Phylogeographic relationships were reconstructed using maximum likelihood and neighbor-joining algorithms in PAUP*b10 [52]. Nodal strength was assessed using 1000 bootstrap replicates. Bayesian statistics were also used to reconstruct phylogeographic relationships in Mr. Bayes v3.4 [53]. Bayesian reconstruction started from a random tree and was run with four heated chains for 1.0 × 10^7^generations, sampling every 1,000 generations. Three runs were conducted and nodal support (posterior probability) was computed from the three runs after stationarity was reached.

Two cytochrome *b *statistical parsimony networks were constructed for the two clades of haplotypes [58]. Haplotypes were connected based on the absolute number of mutational differences and coalescent theory was used to identify ancestral (internal) and derived (tip) relationships [59] in the program TCS v1.21 [60]. Ten mutational steps between any two haplotypes is the maximum difference allowable in order to reconstruct the relationships while meeting the parsimony criterion with 95% probability [58].

The *M*. *rutilus *haplotype network was constructed using 11 haplotypes representing 47 *M*. *rutilus *individuals and also included 17 *M*. *rutilus*-like haplotypes found in 45 individuals identified morphologically as *M*. *gapperi *(introgressants). The *M*. *gapperi *haplotype network was constructed using 18 haplotypes representing 80 *M*. *gapperi *individuals.

### Demographic history and molecular evolution

Variation of cytochrome *b *sequences was used to test for signals of population expansion, to estimate the time since expansion, and to estimate mutation rates. Haplotype diversity (*h*) and nucleotide diversity (π) were estimated [61] separately for *M*. *rutilus*, *M*. *gapperi*, and introgressants in the program Arlequin ver. 3.11 [62]. Differences in levels of diversity were tested using a *t-*test. The frequency distribution of pairwise differences (mismatch distribution) [63] and Fu's F_S _statistic [64,65] were calculated to test for population expansion in Arlequin ver. 3.11. Under the Sudden Expansion Model, pairwise differences will have a unimodal distribution [66]. Values of Fu's F_S _will be negative and indicative of expansion when there is an excess of singleton mutations or a when a gene is under selection [65].

The peak of the unimodal distribution (τ) [66,67] in the mismatch distribution was used to calculate time since expansion in Arlequin. Applying the equation τ = 2 μt where μ is the mutation rate and t is time in generations [66], allows an estimate of time since expansion. Confidence intervals of τ were calculated using parametric bootstrapping [68]. Using the mutation rate of 7.5% per million years [69], and values of τ obtained from mismatch analyses, we estimated time since expansion for *M*. *gapperi*, *M*. *rutilus *and introgressants.

To test for constancy in rates of cytochrome *b *evolution among lineages, we conducted a relative rates test in rrTree v1.1 [70], which calculates synonymous and non-synonymous rates of evolution [71,72]. We also constrained the ML phylogeny to a molecular clock in PAUP*4.0b10 [52] and performed a log likelihood ratio test of log likelihood scores of constrained and unconstrained trees and compared these values to a χ^2 ^distribution.

## Authors' contributions

AMR designed the study, conducted fieldwork, generated the genetic data, examined the morphological variation in the post-palatal bridge, performed all statistical analyses, and wrote the manuscript. MDM provided fellowship support to AMR and contributed to the preparation of the manuscript. JAC assisted in designing the study, supported the fieldwork, and contributed to the preparation of the manuscript. All authors read and approved the final manuscript. Molecular research was conducted in laboratories of JAC and MDM.

## Supplementary Material

Additional file 1 **Specimens examined in this study**. List of specimens and their voucher numbers used in this research. Click here for file

## References

[B1] Dowling TE, Secor C (1997). The role of hybridization and introgression in the diversification of animals. Annu Rev Ecol Syst.

[B2] Seehausen O (2004). Hybridization and adaptive radiation. Trends Ecol Evol.

[B3] Mallet J (2005). Hybridization as an invasion of the genome. Trends Ecol Evol.

[B4] Barton NH, Hewitt GM (1985). Analysis of hybrid zones. Annu Rev Ecol Syst.

[B5] Wilson CC, Bernatchez L (1998). The ghosts of hybrids past: fixation of arctic charr (*Salvelinus alpinus*) mitochondrial DNA in an introgressed population of lake trout (*S. namaycush*). Mol Ecol.

[B6] Redenbach Z, Taylor EB (2002). Evidence for historical introgression along a contact zone between two species of char (Pisces: Salmonidae) in Northwestern North America. Evolution.

[B7] Hewitt GM (2004). Genetic consequences of climatic oscillations in the Quaternary. Philos Trans R Soc Lond, Ser B: Biol Sci.

[B8] Melo-Ferreira J, Boursot P, Suchentrunk F, Ferrand N, Alves PC (2005). Invasion from the cold past: extensive introgression of mountain hare (*Lepus timidus*) mitochondrial DNA into three other hare species in northern Iberia. Mol Ecol.

[B9] Moore WS (1977). An evaluation of narrow hybrid zones in vertebrates. Q Rev Biol.

[B10] Jiggins CD, Naisbit RE, Coe RL, Mallet J (2001). Reproductive isolation caused by colour pattern mimicry. Nature.

[B11] MacPherson AH (1965). The origin of diversity in mammals of the Canadian arctic tundra. Syst Zool.

[B12] Mann DH, Hamilton TD (1995). Late Pleistocene and Holocene paleoenvironments of the North Pacific Coast. Quat Sci Rev.

[B13] Cook JA, Bidlack AL, Conroy CJ, Demboski JR, Fleming MA, Runck AM, Stone KD, MacDonald SO (2001). A phylogeographic perspective on endemism in the Alexander Archipelago. Biol Conserv.

[B14] Canham RP, Cameron DG (1972). Variation in the serum proteins of the red-backed mice *Clethrionomys rutilus *and *C. gapperi *and its taxonomic significance. Can J Zool/Rev Can Zool.

[B15] McPhee EC (1977). Parapatry in *Clethrionomys *: ethological aspects of mutual exclusion in C. gapperi and C. rutilus. PhD thesis.

[B16] Cook JA, Runck AM, Conroy CJ (2004). Historical biogeography at the crossroads of the northern continents: molecular phylogenetics of red-backed voles (Rodentia: Arvicolinae). Mol Phylogen Evol.

[B17] Irwin D, Kocher T, Wilson A (1991). Evolution of the cytochrome b gene of mammals. J Mol Evol.

[B18] Hewitt GM (1996). Some genetic consequences of ice ages, and their role in divergence and speciation. Philos Trans R Soc Lond B Biol Sci.

[B19] Melo-Ferreira J, Boursot P, Randi E, Kryukov A, Suchentrunk F, Ferrand N, Alves PC (2007). The rise and fall of the mountain hare (Lepus timidus) during Pleistocene glaciations: expansion and retreat with hybridization in the Iberian Peninsula. Mol Ecol.

[B20] Hall ER (1981). Mammals of North America.

[B21] Dyke AS, Andrews JT, Clark PU, England JH, Miller GH, Shaw J, Veillette JJ (2002). The Laurentide and Innuitian ice sheets during the Last Glacial Maximum. Quat Sci Rev.

[B22] Loehr J, Worley K, Grapputo A, Carey J, Veitch A, Coltman DW (2006). Evidence for cryptic glacial refugia from North American mountain sheep mitochondrial DNA. J Evol Biol.

[B23] Gyllensten U, Wharton D, Wilson AC (1985). Maternal inheritance of mitochondrial DNA during backcrossing of two species of mice. J Hered.

[B24] Fleming MA, Cook JA (2002). Phylogeography of endemic (*Mustela erminea*) in southeast Alaska. Mol Ecol.

[B25] Carrara PE, Agar TE, Baichtal JF (2007). Possible refugia in the Alexander Archipelago of southeastern Alaska during the late Wisconsin glaciation. Can J Earth Sci/Rev Can Sci Terre.

[B26] Heaton TH, Grady F, Schubert BW, Mead JI, Graham RW (2003). The Late Wisconsin vertebrate history of Prince of Wales Island, Southeast Alaska. Ice Age Cave Faunas of North America.

[B27] Hansen BCS, Engstrom DR (1996). Vegetation history of Pleasant Island, Southeastern Alaska, since 13,000 yr B. P. Quatern Res.

[B28] Mann DH, Ugolini FC (1985). Holocene glacial history of the Lituya District, southeast Alaska. Can J Earth Sci/Rev Can Sci Terre.

[B29] Mann DH, Hamilton TD, Reed KM, Thorson RM (1986). Wisconsin and Holocene glaciations of Southeast Alaska. Glaciation in Alaska: The geologic record.

[B30] Pielou EC (1991). After the Ice Age.

[B31] Goodfriend GA, Gould SJ (1996). Paleontology and chronology of two evolutionary transitions by hybridization in the Bahamian Land Snail *Cerion*. Science.

[B32] Demboski JR, Cook JA (2001). Phylogeography of the dusky shrew, *Sorex monticolus *(Insectivora, Soricidae): insight into deep and shallow history in northwestern North America. Mol Ecol.

[B33] Conroy CJ, Cook JA (2000). Phylogeography of a post-glacial colonizer: *Microtus longicaudus *(Rodentia: Muridae). Mol Ecol.

[B34] Stone KD, Cook JA (2000). Phylogeography of black bears (*Ursus americanus*) from the Pacific Northwest. Can J Zool/Rev Can Zool.

[B35] Stone KD, Flynn RW, Cook JA (2002). Post-glacial colonization of northwestern North America by the forest-associated American marten (*Martes americana*, Mammalia: Carnivora: Mustelidae). Mol Ecol.

[B36] Small MP, Stone KD, Cook JA (2003). American marten (*Martes americana*) in the Pacific Northwest: population differentiation across a landscape fragmented in time and space. Mol Ecol.

[B37] Peacock E (2004). Population, genetic, and behavioral studies of black bears *Ursus americanus *in Southeast Alaska. PhD thesis.

[B38] Remington CL (1968). Suture-zones of hybrid interaction between recently joined biotas. Evol Biol.

[B39] Swenson NG, Howard DJ (2004). Do suture zones exist?. Evolution.

[B40] Swenson NG, Howard DJ (2005). Clustering of contact zones, hybrid zones and phylogeographic breaks in North America. Am Nat.

[B41] MacDonald SO, Cook JA (1996). The land mammal fauna of southeast Alaska. Can Field-Nat.

[B42] Zamudio KR, Savage WK (2003). Historical isolation, range expansion, and secondary contact of two highly divergent mitochondrial lineages in spotted salamanders (*Ambystoma maculatum*). Evolution.

[B43] Cook JA, Dawson NG, MacDonald SO (2006). Conservation of highly fragmented systems: the north temperate Alexander Archipelago. Biol Conserv.

[B44] MacDonald SO, Cook JA (2007). Mammals and Amphibians of Southeast Alaska. Museum of Southwestern Biology Special Publication.

[B45] Merrit JF (1981). Clethrionomys gapperi. Mammalian Species.

[B46] Hall ER, Cockrum EL (1953). A Synopsis of the North American Microtine Rodents. University of Kansas Publications Museum of Natural History.

[B47] Miller SA, Dykes DD, Polesky HF (1988). A simple salting out procedure for extraction DNA from human nucleated cells. Nucleic Acids Res.

[B48] Lyons LA, Laughlin TF, Coopeland NG, Jenkins NA, Womack JE, O'Brien SJ (1997). Comparative anchor tagged sequences (CATS) for integrative mapping of mammalian genomes. Nat Genet.

[B49] Smith MF, Patton JL (1993). The diversification of South American murid rodents: Evidence from mitochondrial DNA sequence data for the akodontine tribe. Biol J Linn Soc.

[B50] Runck AM, Cook JA (2005). Postglacial expansion of the southern red-backed vole (*Clethrionomys gapperi*) in North America. Mol Ecol.

[B51] Bernstein LJ, Abbot BJ (1987). Precipitation of high molecular weight DNA with polyethylene glycol removes contaminating RNA oligonucleotides. BioTechniques.

[B52] Swofford DL (2002). Paup 4.0b10. Phylogenetic Analysis Using Parsimony (*and Other Methods).

[B53] Huelsenbeck JP, Ronquist F (2001). MrBayes: Bayesian inference of phylogeny. Bioinformatics.

[B54] Hasegawa M, Kishino K, Yano T (1985). Dating the human-ape splitting by a molecular clock of mitochondrial DNA. J Mol Evol.

[B55] Posada D, Crandall KA (1998). MODELTEST: testing the model of DNA substitution. Bioinformatics.

[B56] Conroy CJ, Cook JA (1999). MtDNA evidence for repeated pulses of speciation within arvicoline and murid rodents. J Mamm Evol.

[B57] Tamura K, Nei M (1993). Estimation of the number of nucleotide substitution in the control region of mitochondrial DNA in humans and chimpanzees. Mol Biol Evol.

[B58] Templeton AR, Crandall KA, Sing CF (1992). A cladistic analysis of phenotypic associations with haplotypes inferred from restriction endonuclease mapping and DNA sequence data III. Cladogram estimation. Genetics.

[B59] Castelloe J, Templeton AR (1994). Root probabilities for intraspecific gene trees under neutral coalescent theory. Mol Phylogen Evol.

[B60] Clement M, Posada D, Crandall K (2000). TCS: a computer program to estimate gene genealogies. Mol Ecol.

[B61] Nei M (1987). Molecular Evolutionary Genetics.

[B62] Excoffier L, Laval G, Schneider S (2005). Arlequin ver. 3.0: An integrated software package for population genetics data analysis. EVolutionary Bioinformatics Online.

[B63] Rogers A (1992). Genetic evidence for a Pleistocene population explosion. Evolution.

[B64] Rozas J, Sánchez-DelBarrio JC, Messeguer X, Rozas R (2003). DnaSP, DNA polymorphism analyses by the coalescent and other methods. Bioinformatics.

[B65] Fu YX (1997). Statistical test of neutrality of mutations against population growth, hitchhiking and background selection. Genetics.

[B66] Rogers AR, Harpending H (1992). Population growth makes waves in the distribution of pairwise genetic differences. Mol Biol Evol.

[B67] Rogers A (1995). Genetic evidence for a Pleistocene population explosion. Evolution.

[B68] Schneider S, Excoffier L (1999). Estimation of demographic parameters from the distribution of pairwise differences when the mutation rates vary among sites: Application to human mitochondiral DNA. Genetics.

[B69] Fedorov VB, Goropashnaya AV, Jaarola M, Cook JA (2003). Phylogeography of lemmings (*Lemmus*): no evidence for postglacial colonization of Arctic from the Beringian refugium. Mol Ecol.

[B70] Robinson-Rechavi M, Huchon D (2000). RRTree: Relative-rate tests between groups of sequences on a phylogenetic tree. Bioinformatics.

[B71] Li W (1993). Unbiased estimation of the rates of synonymous and nonsynonymous substitutions. Mol Biol Evol.

[B72] Pamilo P, Bianchi N (1993). Evolution of the Zfx and Zfy genes: rates and interdependence between the genes. Mol Biol Evol.

