# Supplementary material for: Historic hybridization and persistence of a novel mito-nuclear combination in red-backed voles (genus Myodes)
Source: BMC Evol Biol. 2009 May 21;9:114. doi: 10.1186/1471-2148-9-114 (PMC2697987; doi:10.1186/1471-2148-9-114)
Supplement: Additional file 1 — Specimens examined in this study. List of specimens and their voucher numbers used in this research. [file 1471-2148-9-114-S1.docx]

**Additional File 1 Specimens examined in this study.**

| Locality | N | Specimens |
| --- | --- | --- |
| 1 Excursion Inlet | 6 | UAM42430**^C^**^,^**^M^**; UAM42431**^C^**^,M^; UAM43449**^C^**^,M^; UAM43450^C,M^; UAM66644**^C^**^,M^; UAM67285**^C^**^,^**^M^**; |
| 2 Mud Bay | 20 | UAM48139^C,M^; UAM50974**^C^**^,^**^M^**^,B^, UAM50975**^C^**^,M,B^; UAM50976**^C^**^,M,B^; UAM50977**^C^**^,M,B^; UAM50978**^C^**^,^**^M^**^,B^; UAM51022^C,M,B^; UAM64161^C,M^; UAM68286^C,M^; UAM68287^C,M^; UAM68470^C,M^; UAM68471^C,M^; UAM68472^C,M^; UAM68473^C,M^; UAM68479^C,M^; UAM68480^C,M^; UAM68481^C,M^; UAM68493^C,M^; UAM68494^C,M^; UAM68495^C,M^ |
| 3 Echo Cove | 20 | UAM50979**^C^**^,M,B^; UAM50980^C,M,B^; UAM50957^C,M,B^; UAM50956^C,M,B^; UAM50960^C,M,B^; UAM50987**^C^**^,M,B^; UAM50988^C,M,B^; UAM50989^C,M,B^; UAM50990^C,M,B^; UAM50991**^C^**^,^**^M^**^,B^; UAM50992^C,M,B^; UAM50993^C,M,B^; UAM50994^C,M,B^; UAM50995^C,M,B^; UAM50996^C,M,B^; UAM50997^C,,B^; UAM50998^C,M,B^; UAM50965**^C^**^,M,B^; UAM50966^C,M^; UAM50963**^C^**^,^**^M^**^,B^ |
| 4 Limestone Inlet | 8 | UAM44609**^C^**^,M^; UAM44608 ^C,M^; UAM44610**^C^**^,^**^M^**^,B^; UAM44611^C,M^; UAM44612**^C^**^,^**^M^**^,B^; UAM44613**^C^**^,M^; UAM44614**^C^**^,M^; UAM44615^C,M^ |
| 5 Cape Fanshaw | 18 | UAM44581**^C^**^,M^; UAM44579**^C^**^,^**^M^**^,B^; UAM44580^C,M^, UAM44582**^C^**^,M^; UAM44585^CM^; UAM44586^C,M^; UAM44587^C,M^; UAM44588^C,M^; UAM44589^C,M^; UAM44590^C,M^; UAM44591**^C^**^,M,B^; UAM44592**^C^**^,M,B^; UAM44593^C,M^; UAM44594^C,M^; UAM44595^C,M^; UAM44597^C,M^; UAM44598**^C^**^,^**^M^**^,B^; UAM44599^C,M^ |
| 6 Patterson River | 13 | UAM48865**^C^**^,^**^M^**^,B^; UAM68454**^C^**^,M^; UAM48867**^C^**^,M,B^; UAM48868**^C^**^,M^, UAM68455^C,M^; UAM48862^C,M,B^; UAM48863**^C^**^,M,B^ ; UAM48864**^C^**^,M,B^; UAM68458**^C,^**^M^; UAM68459**^C^**^,M^; UAM48866**^C^**^,^**^M^**^,B^; UAM48869**^C^**^,M,B^; UAM48870**^C^**^,M,B^ |
| 7 Jap Creek | 10 | UAM48857**^C^**^,^**^M,^**^B^; UAM68435**^C^**^,^**^M^**; UAM68436**^C^**^,M^; UAM68437**^C^**^,M^; UAM68438**^C^**^,M^; UAM68442**^C^**^,M^; UAM68443**^C^**^,M^; UAM68444**^C^**^,M^; UAM68445**^C^**^,M^; UAM68452**^C^**^,M^ |
| 8 Mallard Slough | 28 | UAM50297**^C^**^,^**^M^**^,B^; UAM50298^C,M,B^; UAM50299^C,M^; UAM50300**^C^**^,M,B^; UAM50302^C,M,B^; UAM50303**^C^**^,^**^M^**^,B^; UAM50304**^C^**^,^**^M^**^,B^; UAM50305^C,M,B^; UAM50472^C,M,B^; UAM50478^C,M^; UAM50480^C,M,B^; UAM50481^C,M,B^; UAM50482^C,M^; UAM50483**^C^**^,M,B^; UAM50485^C,M,B^; UAM50487^C,M,B^; UAM50488^C,M,B^; UAM50489**^C^**^,M,B^; UAM50490^C,M,B^; UAM5049^C,M,B^; UAM50497^C,M,B^; UAM51018^C,M,B^; UAM51019^C,M^; UAM51020**^C^**^,M,B^; UAM51024**^C^**^,M,B^; UAM68271^C,M^; UAM68272^C,M^; UAM68274^C,M^; |
| 9 Stikine River | 20 | UAM20793**^C^**^,M^; UAM20794**^C^**^,M,B^; UAM20796**^C^**^,M^; UAM20797**^C^**^,M,B^;UAM20798^C,M,B^; UAM20799^C,M^; UAM20806**^C^**^,M^; UAM20811^C,M^; UAM20812^C,M^; UAM20815^C,M^; UAM29981**^C^**^,^**^M^**^,B^; UAM29982^C,M^; UAM29983^C,M^; UAM29984**^C^**^,^**^M^**^,B^; UAM29985^C,M^; UAM29986^C,M,B^; UAM29987^C,M,B^; UAM29988^C,M^; UAM29989**^C^**^,M,B^; UAM30708^C,M^ |
| 10 Berg Bay | 5 | UAM50293**^C^**^,^**^M^**^,B^, UAM50294**^C^**^,^**^M^**^,B^; UAM50295**^C^**^,M^; UAM50296**^C^**^,M,B^; UAM68414**^C^**^,M,^ |
| 11 Tyee | 29 | UAM51962**^C^**^,M,B^; UAM52176**^C^**^,M,B^; UAM52178^C,M,B^; UAM64037**^C^**^,M,B^; UAM64038^C,M,B^; UAM69919^C,M^; UAM69920^C,M^; UAM69221^C,M^; UAM69922**^C^**^,^**^M^**; UAM69923^C,M^; UAM69925**^C^**^,M^;UAM69930^C,M^; UAM69948**^C^**^,^**^M^**^,B^; UAM69949^C,M^; UAM69954^C,M,B^; UAM69955**^C^**^,^**^M^**^,B^; UAM69956**^C^**^,^**^M^**^,B^; UAM69957**^C^**^,M,B^; UAM69958^C,M,B^; UAM69972^C,M^; UAM69973^C,M^; UAM69987^C,M^; UAM69988^C,M^; UAM69989**^C^**^,M^; UAM69990^C,M^; UAM69991^C,M^; UAM70017^C,M^; UAM70018^C,M^; UAM70020^C,M^ |
| 12 Reflection Lake | 17 | UAM71045**^C^**^,M,B^; UAM71046**^C^**^,M,B^; UAM71047^C,M,B^; UAM71048**^C^**^,M,B^; UAM71049^C,M,B^; UAM71050**^C^**^,M,B^; UAM71064**^C^**^,^**^M^**^,B^ UAM71065**^C^**^,^**^M^**^,B^; UAM71108**^C^**^,M,B^; UAM71087^C,^**^M^**^,B^; UAM71094**^C^**^,^**^M^**^,B^; UAM71107^C,M,B^; UAM71119**^C^**^,^**^M^**^,B^;UAM71130^C,M,B^; UAM71143**^C^**^,^**^M^**^,B^; UAM71144^C,M,B^; UAM71173^C,M,B^; |
| 13 Unuk River | 31 | UAM23474^C,M,B^; UAM23488^C,M,B^;UAM23494^C,M,B^; UAM23495^C,M,B^; UAM23505^C,M,B^; UAM23531**^C^**^,M,B^; UAM23532^C,M,B^; UAM23533^C,M,B^; UAM23547^C,M,B^; UAM23549^C,M,B^; UAM23551**^C^**^,^**^M^**^,B^; UAM23553^C,M,B^; UAM23562^C,M,B^; UAM23563^C,M,B^; UAM23573^C,M,B^; UAM23588^C,M,B^; UAM23591**^C^**^,M,B^; UAM23603**^C^**^,M,B^; UAM23571^C,M^; UAM23589^C,M,B^; UAM46943^C,M^; UAM23602^C,M,B^; UAM23493^C,M,B^; UAM23596^C,M,B^; UAM23917^C,M,B^; UAM23918^C,M,B^; UAM23919^C,M,B^; UAM23920**^C^**^,^**^M^**^,B^; UAM23921^C,M^; UAM23922^C,M^; UAM62591^C,M^; |
| 14 Chickamin River | 15 | UAM10360^C,M,B^; UAM10361^C,M,B^; UAM10363^C,M,B^; UAM18724^C,M,B^; UAM18726^C,M,B^; UAM23923^C,M,B^; UAM23924^C,M,B^; UAM23925**^C^**^,^**^M,^**^B^; UAM23926**^C^**^,^**^M^**^,B^; UAM23927**^C^**^,M,B^; UAM23928**^C^**^,M,B^; UAM23929^C,M,B^; UAM23930**^C^**^,M,B^; UAM23931^C,M,B^; UAM76251^C,M,B^ |
| 15 Hut Point | 16 | UAM71420**^C^**^,^**^M^**^,B^; UAM71421**^C^**^,M,B^; UAM71422**^C^**^,M,B^; UAM71423**^C^**^,^**^M^**^,B^; UAM71424**^C^**^,M,B^; UAM71425^C,M,B^; UAM71426^C,M,B^; UAM71427**^C^**^,M^; UAM71428**^C^**^,M,B^; UAM71435^C,M,B^; UAM71436^C,M,B^; UAM71437^C,M,B^; UAM71438^C,M,B^; UAM71436**^C^**^,^**^M^**^,B^; UAM71464**^C^**^,M,B^; UAM71462**^C^**^,^**^M^**^,B^; |
| 16 Ledge Point | 7 | UAM71412**^C^**^,M,B^; UAM71413**^C^**^,^**^M^**^,B^; UAM71414^C,M,B^; UAM71415**^C^**^,M,B^; UAM71416^C,M,B^; UAM71417**^C^**^,^**^M^**^,B^; UAM71457**^C^**^,M,B^ |
| 17 N Rudyerd Bay | 17 | UAM71308**^C^**^,M,B^; UAM71309**^C^**^,M,B^; UAM71310**^C^**^,^**^M^**^,B^; UAM71311**^C^**^,M,B^; UAM71312^C,M^; UAM71042^C,M^; UAM71352^C,M^; UAM71353^C,M^; UAM71354**^C^**^,M,B^; UAM71355^C,M^; UAM71356^C,M,B^; UAM71373^C,M,B^; UAM71374**^C^**^,^**^M^**^,B^; UAM71375^C,M^; UAM71376^C,M,B^; UAM71377^C,M,B^; UAM71451^C,M,B^ |
| 18 Pt. Louise | 19 | UAM71295**^C^**^,^**^M^**^,B^; UAM71297^C,M,B^; UAM71298^C,M,B^; UAM71299**^C^**^,M,B^; UAM71300**^C^**^,^**^M^**^,B^; UAM71301^C,M,B^; UAM71302^C,M^; UAM71303**^C^**^,^**^M^**^,B^; UAM71304^C,M,B^; UAM71396^C,M,B^; UAM71397**^C^**^,M,B^; UAM71398^C,M^; UAM71345^C,M,B^; UAM71346^C,M,B^; UAM71347**^C^**^,M,B^; UAM71348^C,M^; UAM71369^C,M^; UAM71370^C,M^; UAM71449^C,M,B^ |
| 19 Gwent Cove | 20 | UAM58071**^C^**^,M^; UAM70171^C,M,B^; UAM70173^C,M.B^; UAM70174^C,M,B^; UAM70175^C,M,B^; UAM70276**^C^**^,^**^M^**^,B^; UAM70280**^C^**^,M,B^; UAM70288**^C^**^,^**^M^**^,B^; UAM70289**^C^**^,M,B^; UAM70297^C,M,B^; UAM70257^C,M,B^; UAM70272^C,M,B^; UAM70273^C,M,B^; UAM70274^C,M^; UAM70275^C,M,B^; UAM70297^C,M,B^; UAM70298^C,M,B^; AF25578**^C^**^,^^M^; AF26600^C,M,B^; AF26600^C,M,B^; SOM2264^B^ |
| 20 Duck Point | 26 | UAM64034^C,M,B^; UAM64039**^C^**^,^**^M^**^,B^; UAM70022**^C^**^,M^; UAM70023**^C^**^,M^; UAM70025**^C^**^,M;B^; UAM70025**^C^**^,M^; UAM70029**^C^**^,^**^M^**; UAM70051**^C^**^,M^; UAM70052^C,M^; UAM70053**^C^**^,M^; UAM70054^C,M^; UAM70055**^C^**^,M^; UAM70056^C,M^; UAM70057^C,M^; UAM70058^C,M^; UAM70059^C,M^; UAM70060^C,M^; UAM70064^C,M^; UAM70067**^C^**^,M^; UAM70069^C,M^; UAM70070**^C^**^,M^; UAM70026^C,M^; UAM70028^C,M^; UAM70065^C,M^; UAM70066^C,M^; UAM70068^C,M^ |
| 21 Union Bay | 25 | UAM23753**^C^**^,M^; UAM23754**^C^**^,^**^M^**^,B^; UAM23755**^C^**^,^**^M^**^,B^; UAM23756**^C^**^,M,B^; UAM23757^C,M,B^; UAM23758^C,M^; UAM23759**^C^**^,M^; UAM23760^C,M^; UAM23761^C,M^; UAM23762^C,M,B^; UAM23772^C,M,B^; UAM23790^C,M,B^; UAM23791^C,M,B^; UAM23792^C,M^; UAM23793^C,M^; UAM23794^C,M^; UAM23795^C,M^; UAM23797^C,M^; UAM23798^C,M^; UAM23799^C,M,B^; UAM23800^C,M,B^; UAM23801^C,M^; UAM23802^C,M^; UAM23789^C,M,B^; UAM46989^B^ |
| 22 Bond Bay | 24 | UAM51131^C,M,B^; UAM51132^C,M,B^; UAM51133^C,M,B^; UAM51134^C,M,B^; UAM51135^C,M,B^; UAM51136^C,M,B^; UAM51140**^C^**^,M,B^; UAM51141^C,M,B^;UAM51142^C,M,B^; UAM51143^C,M,B^; UAM51145^C,M,B^; UAM51146^C,M,B^; UAM51147^C,M,B^; UAM51148^C,M,B^; UAM51149^C,M,B^; UAM51150**^C^**^,^**^M^**^,B^; UAM51152^C,M,B^; UAM51155^C,M,B^; UAM51156^C,M,B^; UAM51157**^C^**^,^**^M^**^,B^; UAM51158**^C^**^,^**^M^**^,B^; UAM51159**^C^**^,M,B^; UAM51160^C,M,B^; UAM51162^C,M,B^ |
| 23 Wrangell Island | 36 | UAM50109^C,M,B^; UAM50110^C,M,B^; UAM50111^C,M,B^; UAM50112^C,M,B^; UAM50113^C,M,B^; UAM50115^C,M,B^; UAM50116^C,M,B^; UAM50117^C,M,B^; UAM64033^C,M,B^; UAM64041^C,M,B^; UAM69774^C,M,B^; UAM69776^C,M,B^; UAM69777^C,M,B^; UAM69778**^C^**^,^**^M^**^,B^; UAM69779**^C^**^,^**^M^**^,B^; UAM69781**^C^**^,M,B^; UAM69784**^C^**^,M,B^; UAM69785**^C^**^,^**^M^**^,B^; UAM69787^C,M,B^; UAM69788^C,M,B^; UAM69789^C,M,B^; UAM69790^C,M^; UAM69794^C,M^; UAM69795^C,M^; UAM69797^C,M,B^; UAM69798^C,M,B^; UAM69799^C,M,B^; UAM69800^C,M,B^; UAM69803^C,M,B^; UAM69804^C,M,B^; UAM69805^C,M,B^; UAM69807^C,M,B^; UAM69808^C,M,B^; UAM69811^C,M,B^; UAM69823^C,M,B^; UAM69818^C,M,B^ |
| 24 Etolin Island | 34 | UAM20640^C,M,B^; UAM20649^C,M,B^; UAM41646^C,M,B^; UAM41648^C,M,B^; UAM41649^C,M,B^; UAM41885^C,M,B^; UAM41886^C,M,B^; UAM41887^C,M,B^; UAM41889^C,M,B^; UAM41890^C,M,B^; UAM41891^C,M,B^; UAM43129^C,M,B^; UAM43132^C,M,B^; UAM43133^C,M,B^; UAM52177^C,M^; UAM59163**^C^**^,M,B^; UAM69835^C,M^; UAM69836^C,M^; UAM69837**^C^**^,^**^M^**^,B^; UAM69838^C,M^; UAM69844^C,M^; UAM69845**^C,^**^M,B^; UAM69848^C,M,B^; UAM69849**^C^**^,M,B^; UAM69853**^C^**^,^**^M^**^,B^; UAM69854^C,M^; UAM69859^C,M,B^; UAM6987^C,M,B^; UAM69872^C,M^; UAM69873**^C^**^,M^; UAM69874^C,M^; UAM69879^C,M,B^; UAM69880^C,M,B^; UAM69885^C,M^; |
| 25 Revillagigedo Is. | 12 | UAM71477^C,M,B^; UAM71478**^C^**^,M,B^; UAM71486^C,M,B^; UAM71494^C,M,B^; UAM71495**^C^**^,^**^M^**^,B^; UAM71496^C,M,B^; UAM71497**^C^**^,^**^M^**^,B^; UAM71498^C,M,B^; UAM71499^C,M,B^; AF29545**^C^**^,M^; AF29588^C,M^; AF29589**^C^**^,M^ |
| 26 Revillagigedo Is. | 9 | UAM71217**^C^**^,^**^M^**^,B^; UAM71218**^C^**^,M,B^; UAM71219**^C^**^,M,B^; UAM71220^C,M^; UAM71221**^C^**^,M,B^; UAM71249**^C^**^,^**^M^**^,B^; UAM71250^C,M^; UAM71251^C,M^; UAM71261^C,M,B^ |
| Interior Alaska | 46 | UAM24610^B^; UAM24616^B^; UAM24617^B^; UAM24618^B^; UAM24619^B^; UAM24620^B^; UAM24624^B^; UAM24637^B^; UAM24640^B^; UAM24644^B^; UAM24647^B^; UAM24650^B^; UAM24651^B^; UAM24653^B^; UAM24654^B^; UAM24657^B^; UAM24659^B^; UAM24661^B^; UAM24662^B^; UAM24663^B^; UAM24664^B^; UAM34137^B^; UAM34164^B^; UAM34246^B^; UAM34247^B^; UAM34250^B^; UAM50546^B^; UAM50593^B^; UAM50594^B^; UAM51526^B^; UAM51530^B^; UAM51460^B^; UAM51468^B^; UAM51474^B^; UAM51478^B^; UAM51484^B^; UAM51488^B^; UAM51495^B^; UAM51496^B^; UAM51503^B^; UAM51504^B^; UAM51509^B^; UAM51510^B^; UAM51513^B^; UAM51521^B^; UAM51527^B^ |
| Minnesota | 18 | UAM59437^B^; UAM59438^B^; UAM59439^B^; UAM59458^B^; UAM59482^B^; UAM59495^B^; UAM59496^B^; UAM59501^B^; UAM59508^B^; UAM59513^B^; UAM59518^B^; UAM59521^B^; UAM59523^B^; UAM59524^B^; UAM59534**^C^**^,^**^M^**^,B^; UAM59548^B^; UAM59549^B^; UAM59468^B^ |

Additional file 1 legend: Sampling localities with locality number corresponding to Figure 1& 6 and Table 1. Number of individuals examined for morphological and/or molecular data (N). Data types are indicated by C (cytochrome *b* gene), M (nuclear locus MYH6), and B (morphology of the post-palatal bridge). Bolded letters indicate that the gene was sequenced.
